# Maternal Quercetin Consumption during Pregnancy May Help Regulate Total Cholesterol/HDL-Cholesterol Ratio without Effect on Cholesterol Levels in Male Progeny Consuming High-Fat Diet

**DOI:** 10.3390/nu13041242

**Published:** 2021-04-09

**Authors:** Masakatsu Takashima, Wataru Tanaka, Hiroki Matsuyama, Hayato Tajiri, Hiroyuki Sakakibara

**Affiliations:** Graduate School of Agriculture, University of Miyazaki, 1-1 Gakuen-kibanadai Nishi, Miyazaki 889-2192, Japan; hurricane600km@gmail.com (M.T.); gc14026@student.miyazaki-u.ac.jp (W.T.); gc15047@student.miyazaki-u.ac.jp (H.M.); gc16021@student.miyazaki-u.ac.jp (H.T.)

**Keywords:** high-fat diet–induced obesity, mice, progeny, quercetin, total cholesterol/HDL-cholesterol ratio

## Abstract

Quercetin has been shown to have anti-obesity effects, but it is unknown whether these effects can be transmitted from mothers to their progeny. In this study, we investigated whether maternal quercetin consumption during pregnancy has a protective effect on high-fat diet–induced hyper lipid levels and overweight in progeny. Female mice consumed a control diet or a diet containing 1.0% quercetin during breeding. The male progeny were then divided into four groups that were (1) sacrificed at postnatal day 3; (2) born to dams fed the control diet and also fed the control diet (C-C), (3) born to dams fed the control diet and then fed a 30% high-fat diet (C-HF), or (4) born to dams fed the Q-diet and then fed the HF diet (Q-HF). Maternal consumption of quercetin did not affect body weight or blood lipid parameters in either dams or neonates at postnatal day 3. After 13 weeks, the Q-HF group exhibited greater body and liver weights, and higher blood cholesterol levels than the C-HF group. However, the total cholesterol/ high density lipoprotein (HDL)-cholesterol ratios in the Q-HF and C-C groups remained similar. In conclusion, maternal quercetin consumption does not appear to protect the next generation from high-fat diet–induced hyper cholesterol level in the blood and liver, and consequently overweight, but may help regulate the total cholesterol/HDL-cholesterol ratio.

## 1. Introduction

The World Health Organization (WHO) classifies overweight and obesity based on body mass index (BMI, in kg/m^2^) with the ranges of 18.5–24.9 for normality, 25.0–29.9 for overweight, and ≥30.0 for obesity for adults [1,2]. The BMI values are age-independent and the same for both sexes, but it is important to know that BMI may not correspond to the same degree of fatness in different populations due to differences in body proportions [2]. For example, Polynesians tend to have a lower fat percentage than Caucasian Australians at an identical BMI [3]. Anyway, analysis of life insurance data indicates that obesity is associated with increased mortality, owing to highly elevated risks of adverse health outcomes such as type 2 diabetes mellitus [4,5]. It is estimated that the combined medical costs associated with treatment of obesity-related diseases will increase by $48–66 billion/year in the USA and £1.9–2.0 billion/year in the UK by 2030 [6]. Therefore, implementing effective policies to promote healthier weight may yield economic benefits.

Obesity occurs when the body’s energy balance is positive; for example, when energy intake exceeds energy expenditure [7]. Additionally, dietary patterns have changed recently, and daily consumption of a high-fat diet has been recognized as leading to dyslipidemia and obesity [8]. Management of daily food consumption is thus important for controlling weight. On the other hand, studies have reported that many foods contain ingredients with no nutritional value that protect against obesity by inhibiting lipid absorption and accumulation or promoting energy combustion. One of these ingredients, quercetin (and its glucosides, which are major flavonoids) is considered a candidate for combatting obesity, as it was recently reported to exhibit anti-obesity activity and to regulate lipid metabolism using mice models [9,10,11]. Our group has also demonstrated that the quercetin glucoside, monoglucosyl rutin, has anti-obesity effects using mice models [12]. Daily consumption of quercetin glucosides inhibits diet-induced visceral fat accumulation by regulating the secretion of gastric inhibitory polypeptide (GIP), thereby preventing excess weight gain. Moreover, daily consumption of quercetin-rich onion has been reported to be beneficial for preventing obesity by a randomized, double-blind, placebo-controlled, parallel-group study [13].

In recent decades, the Developmental Origins of Health and Disease (DOHaD) theory has emerged, promoting vigorous research that combines experimental, clinical, epidemiological, and public health approaches [14]. The DOHaD theory postulates that exposure to certain environmental influences during critical periods of development and growth may have significant consequences on an individual’s short- and long-term health [15,16]. For example, from a dietary perspective, a maternal high-fat diet increases obesity susceptibility in offspring due to prenatal programming, resulting in differential programming effects on the offspring’s microbiota and reduced physical activity and energy expenditure later in life [17,18]. A maternal diet containing low-quality protein increases plasma aspartic acid, glutamic acid, histidine, and cystathionine levels and decreases plasma lysine levels in male offspring [19]. Maternal folic acid insufficiency during early pregnancy promotes neoplasm progression in offspring through modulation of DNA methylation [20]. A maternal high-fructose diet programs male offspring to have differential vulnerability to developing hypertension in response to a high-fructose or high-fat diet post-weaning [21]. Thus, much is known about the effects of exposure to essential nutrients during critical periods of development and growth via maternal consumption; however, our knowledge regarding non-essential dietary ingredients such as flavonoids remains incomplete. Wu et al. reported that maternal quercetin consumption in obese dams during gestation and lactation reduces birth weight and postnatal body weight gain in the offspring [22]. In this study, therefore, we focused on quercetin as a representative flavonoid and investigated whether maternal quercetin consumption during pregnancy protects progeny from high-fat diet–induced hyper lipid levels and overweight, to evaluate the possibility of recommending maternal quercetin consumption to promote healthier weight in the next generation.

## 2. Materials and Methods

### 2.1. Chemicals

Quercetin (≥95%) was purchased from Sigma-Aldrich (St. Louis, MO, USA). Cellulose, α-cornstarch, β-cornstarch, and sucrose were purchased from Oriental Yeast Co., Ltd. (Tokyo, Japan). Casein, soybean oil, L-cystine, and t-butylhydroquinone were purchased from Wako Pure Chemical Industries, Ltd. (Osaka, Japan). The vitamin mixture (AIN-93-VX), mineral mixture (AIN-93G-Mix), and lard were purchased from MP Biomedicals, LLC (Irvine, CA, USA). All reagents used were of the highest grade available.

### 2.2. Animal Experiments

#### 2.2.1. Institutional Approval of the Study Protocols

All animal procedures were approved by the Institutional Animal Care and Use Committee of the University of Miyazaki, Japan (Approved No. 2018-008). This study was conducted in accordance with the Japanese Law for the Humane Treatment and Management of Animals (Law No. 105, 1973), which defines animal experimentation as the use of animals for scientific purposes, with consideration of the 3 Rs.

#### 2.2.2. Animals

Eight-week-old ICR mice (male, n = 16; female, n = 48) were procured from an approved vendor (Japan SLC, Shizuoka, Japan). The animals were housed in cages maintained in a room with controlled environmental conditions (temperature, 23 ± 2 °C; humidity, 55 ± 10%) under a 12-h dark/light cycle (light period: 09:00 AM to 09:00 PM) with free access to deionized water and powdered AIN-93G diet (control diet; C-diet), as shown in Table 1.

#### 2.2.3. Breeding

The breeding was carried out using a modified version of the harem method described previously [23,24]. Briefly, after 1 week of acclimatization, half of the female mice were switched from the C-diet to powdered AIN-93G diet containing 1.0% quercetin (Q-diet; Table 1). The amount of quercetin included in the diet was selected based on the results from Yamaki and Takahashi [25], which showed that this quantity does not affect body weight when mice are allowed *ad libitum* consumption. After 3 days of Q-diet consumption, a single stud male was introduced into each breeding cage containing 3 females (n = 8 cages per group). The presence of a vaginal plug was designated as gestational day 0. Plug-positive female mice were moved into individual cages (W 235 mm × L165 mm × H125 mm) with paper bedding (Alpha-dri Certified, EPS Ekishin Co., Tokyo, Japan) and nesting materials (Envero-dri, EPS Ekishin Co., Tokyo, Japan). On the other hand, every sire was euthanized with phlebotomy under the deep anesthesia using isoflurane (2.0%). Cages containing pregnant dams were visually observed twice daily after gestational day 19 for the presence of new litters. The day that offspring appeared was considered postnatal day 0. Among C-diet dams and Q-diet dams, in total nineteen and eighteen females could normally give birth, respectively.

#### 2.2.4. Experimental Design

**Figure 1:**
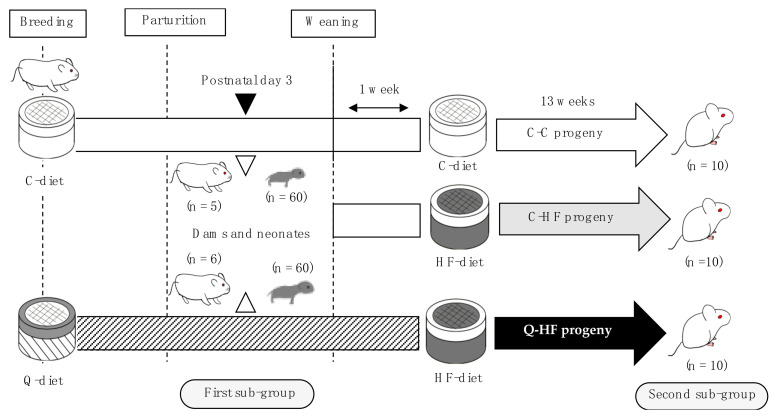
Schematic of the experimental design. After acclimatization on the control diet (C-diet), the quercetin groups were switched to a diet containing 1.0% quercetin (Q-diet). Breeding was carried out after 3 days on the Q-diet. The C-diet dams and their neonates, as well as the Q-diet dams and their neonates, were sacrificed on postnatal day 3 (first sub-group). Furthermore, 20 male progeny born to C-diet dams and 10 male progeny born to Q-diet dams were divided into three groups: male progeny of dams fed the C-diet that were also fed the C-diet (C-C), progeny of dams fed the C-diet that were fed a 30% high-fat (HF) diet (C-HF), and progeny of dams fed Q-diet that were fed a HF-diet (Q-HF). The animals were permitted free access C-diet or HF-diet for 13 weeks.

In this study, we designed the experiments as shown in Figure 1. We could not exactly control the date of their birth, indicating that there was the lag period between the first and last birth. Therefore, the neonates with similar date of birth were employed for the second sub-group, and the others were for the first sub-group. Briefly, the first sub-group, which was composed of C-diet dams (*n* = 5) and their neonates (*n* = 60), as well as Q-diet dams (*n* = 6) and their neonates (*n* = 60), was sacrificed at postnatal day 3. The remaining neonates were weaned at 4 weeks after birth, and male animals were separated for the second sub-group, which was composed of 20 male progeny born to C-diet dams and 10 male progeny born to Q-diet dams, which was weighed and then randomly divided into 5 mice per cage (W335 mm × L225 mm × H135 mm), mixing progeny from different litters. After 1 week of acclimatization consuming the same diet during the breeding, the cages were divided into three groups, as shown in Figure 1: progeny of mothers fed the C-diet that were also fed the C-diet (C-C), progeny of mothers fed the C-diet that were fed a 30% high-fat (HF) diet (C-HF), and progeny of mothers fed the Q-diet that were fed the HF diet (Q-HF). The female progeny and the remining male progeny were euthanized with phlebotomy under the deep anesthesia using isoflurane. The animals were permitted free access to deionized water and either the C-diet or the HF-diet for 13 weeks. During the experimental period, body weight and food consumption were measured three times per week.

#### 2.2.5. Sample Collections

The dams and pups in the first sub-group were fasted for 6 h on postnatal day 3. Blood was drawn from the abdominal vein under anesthesia with isoflurane (1.5%) from the dams and collected in EDTA-coated tubes (Microtainer Tube with EDTA and Microgard Closure, BD, Mississauga, ON, USA). Next, the neonates were anesthetized with isoflurane (1.5%) and decapitated. Trunk blood from neonates born to the same dam was collected in a single EDTA-coated tube. The blood samples were then centrifuged at 2000 × *g* for 90 s at 15 °C, and the plasma fraction was stored at −80 °C until analysis.

For the second sub-group after consumption of C-diet or HF-diet for 13 weeks, blood was drawn from the abdominal vein under anesthesia with isoflurane (1.5%) after fasting for 6 h, and collected in EDTA-coated tubes. The plasma fraction was separated by centrifugation at 2000 × *g* for 90 s at 15 °C and stored at −80 °C until analysis. The liver, kidney, spleen, visceral fat (epididymal fat + perirenal fat), heart, and lung were weighed by operators, who were not informed about the kind of treatment each animal had consumed. One liver section was flash-frozen in liquid nitrogen and stored at −80 °C for later lipid analysis.

### 2.3. Biochemical Parameters

#### 2.3.1. Blood Biochemistry and Adipokines

Plasma biochemical parameters [total cholesterol, high density lipoprotein (HDL)-cholesterol, triglycerides, total protein, and glucose] were analyzed using a Dri-Chem 4000 v chemistry analyzer (Fujifilm Co., Tokyo, Japan) and an individual cartridge slide. Non–HDL-cholesterol amounts were calculated using the following formula:

Non–HDL-cholesterol = total cholesterol − HDL-cholesterol

Detection and quantification of plasma leptin, GIP, and monocyte chemotactic protein-1 (MCP-1) levels in the second sub-group were carried out using an MMHMAG-44K mouse metabolic hormone panel multiplex biometric enzyme-linked immunosorbent assay (Millipore, Billerica, MA, USA) according to the manufacturer’s instructions. The results were analyzed using Luminex MAGPIX system (Millipore).

#### 2.3.2. Hepatic Lipid Analysis

Hepatic lipid levels in the second sub-group were determined as we described previously [12]. Briefly, 200-mg liver samples were homogenized with 1 mL of 50 mM sodium acetate. Subsequently, 6 mL of chloroform–methanol (2:1 [vol/vol]) was added, and the mixture was incubated at 40 °C for 30 min. A 500-µL aliquot of the organic phase was dried using a centrifugal concentrator (CC-105; Tomy Seiko Co., Ltd., Tokyo, Japan). The residues were dissolved in 80 µL of 10% Triton X-100 containing isopropyl alcohol, and triglycerides, total cholesterol, and phospholipid levels were analyzed using individual test kits purchased from Wako Pure Chemical Industries, Ltd.

### 2.4. Statistical Analysis

Data are presented as the mean ± standard deviation (SD). Statistical analyses were conducted using StatView for Windows (version 5.0, SAS Institute, Cary, NC, USA). Statistical significance among groups was determined using two-way analysis of variance (ANOVA), followed by Fisher’s PLSD *post hoc* test. To make comparisons within the groups, alpha value was set to 0.05.

## 3. Results

### 3.1. Effects of Maternal Quercetin Consumption on Neonatal Biological and Blood Parameters

During breeding, dams consumed similar amounts of the C-diet and Q-diet (Table 2). The quercetin consumption of the dams fed the Q-diet was calculated to be 77 ± 10 mg/mice/day. There was no difference between the body weights of dams fed the Q-diet and their neonates and those of the dams fed the C-diet and their neonates at postnatal day 3 (Table 2).

**Figure 2:**
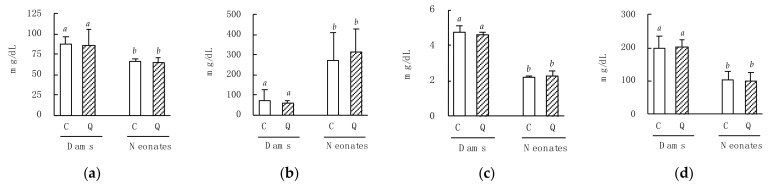
Effects of maternal quercetin consumption during pregnancy on plasma parameters in dams and neonates. After acclimatization on the control (C) diet, the quercetin groups were switched to a diet containing 1.0% quercetin (Q). Breeding was carried out after 3 days on the Q-diet. The C-diet dams and their neonates, as well as the Q-diet dams and their neonates, were sacrificed on postnatal day 3, and then their plasma parameters were analyzed: (**a**) total cholesterol; (**b**) triglycerides; (**c**) total protein; (**d**) glucose. Data are shown as the mean ± SD. Different superscripts (*a*, *b*) indicate significant differences (*p* < 0.05).

Maternal quercetin consumption during pregnancy did not change maternal plasma parameters analyzed on postnatal day 3 (Figure 2). There was also no difference in these parameters between neonates born to dams that consumed the C-diet or the Q-diet. However, total cholesterol, total protein, and glucose levels were significantly lower in the neonates than in their dams. Additionally, triglyceride levels were significantly higher in neonates than in their dams.

### 3.2. Effects of Maternal Quercetin Consumption on Biological Parameters in Progeny Fed a HF Diet

#### 3.2.1. Effects on Food Intake, Body Weight, and Organ Weight

**Figure 3:**
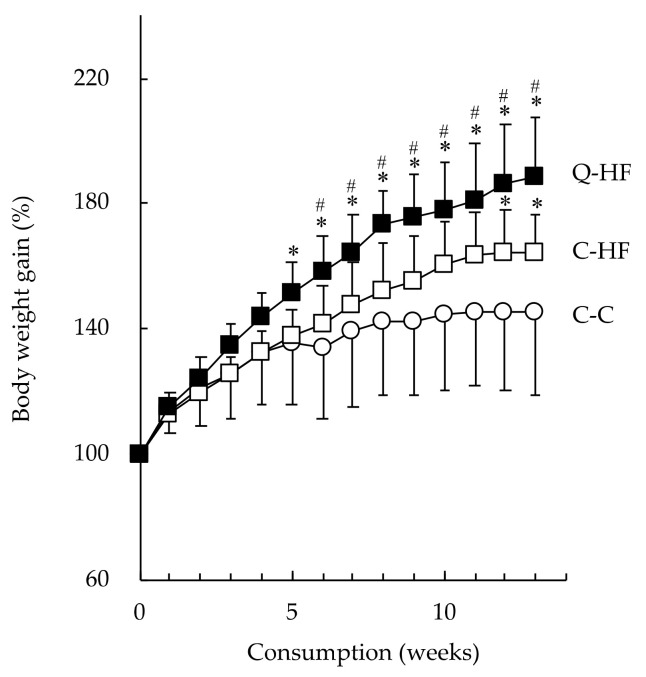
Effects of maternal quercetin consumption on body weight gain in progeny fed a HF diet. Male progeny of dams fed a control diet (C) or a diet containing 1.0% quercetin (Q) were divided into three groups: progeny of dams fed the C-diet that were also fed the C diet (C-C, ○), progeny of dams fed the C-diet that were fed a 30% high-fat (HF) diet (C-HF, □), and progeny of dams fed Q-diet that were fed a HF diet (Q-HF, ■). The animals were permitted free access to deionized water and the C-diet or HF-diet for 13 weeks. Data are shown as the mean ± SD (n = 10). Two-way ANOVA analysis were performed, and Fisher’s PLSD *post hoc* test was applied if ANOVA *p* values were less than 0.05. * vs. the C-C group, # vs. the C-HF group (*p* < 0.05).

After 6 weeks, the mice in the C-HF group exhibited greater body weight gain compared with the C-C group, and this difference was significant at week 12 (Figure 3). The body weights of the progeny in the Q-HF group were higher at 3 weeks, and significantly higher at 5 weeks, than those of the C-C group. After 13 weeks, the body weights in both the C-HF and Q-HF groups were significantly greater than those of the C-C group, by 16.1% and 30.6%, respectively (Table 3). Throughout the experimental period, the progeny in the C-C, C-HF, and Q-HF groups had similar dietary energy intakes (20.1, 20.4, and 20.9 kcal/mouse/day, respectively). Visceral fat weight increased significantly in both the C-HF and the Q-HF group, but there was no difference between these two groups. Liver weight increased significantly in the Q-HF group, but not in the C-HF group. Kidney, spleen, heart, and lung weights were unchanged in all groups.

#### 3.2.2. Effects on Blood Biochemical Parameters

**Figure 4:**
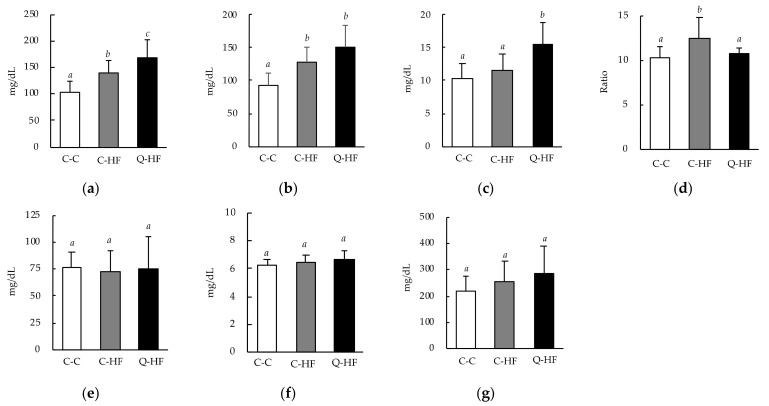
Effects of maternal quercetin consumption on blood biochemical parameters. Male progeny of dams fed a control diet (C) or a diet containing 1.0% quercetin (Q) were divided into three groups: progeny of dams fed the C-diet that were also fed the C-diet (C-C), progeny of dams fed the C-diet that were fed a 30% high-fat (HF) diet (C-HF), and progeny of dams fed Q-diet that were fed a HF-diet (Q-HF). The animals were permitted free access to deionized water and the C-diet or HF-diet for 13 weeks. Data are shown as the mean ± SD (*n* = 10). Two-way ANOVA analysis were performed, and Fisher’s PLSD *post hoc* test was applied if ANOVA *p* values were less than 0.05. Different superscripts (*a*, *b*, *c*) indicate significant differences (*p* < 0.05). (**a**) Total cholesterol. (**b**) non–HDL-cholesterol. (**c**) HDL-cholesterol. (**d**) Total cholesterol/HDL-cholesterol ratio. (**e**) Triglycerides. (**f**) Total protein. (**g**) Glucose.

At week 13, the plasma total cholesterol and non–HDL-cholesterol levels were significantly higher in both the C-HF and Q-HF groups than in the C-C group (Figure 4a,b). The plasma HDL-cholesterol level was significantly increased in the Q-HF group, but not in the C-HF group (Figure 4c). The total cholesterol/HDL cholesterol ratio was significantly increased in the C-HF group, but not in the Q-HF group (Figure 4d). There were no differences in plasma triglycerides, total protein, or glucose levels among the groups (Figure 4e–g).

#### 3.2.3. Effects on Blood Metabolic Hormones and Myokines

**Figure 5:**
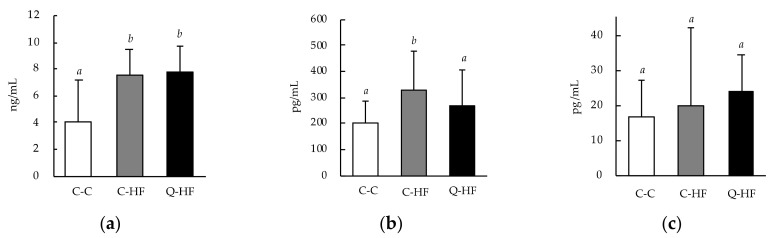
Effects of maternal quercetin consumption on blood metabolic hormones and myokines. Male progeny of dams fed a control diet (C) or a diet containing 1.0% quercetin (Q) were divided into three groups: progeny of dams fed the C-diet that were also fed the C diet (C-C), progeny of dams fed the C-diet that were fed a 30% high-fat (HF) diet (C-HF), and progeny of dams fed Q-diet that were fed a HF-diet (Q-HF). The animals were permitted free access to deionized water and the C-diet or HF-diet for 13 weeks, and then their plasma parameters were analyzed: (**a**) leptin; (**b**) gastric inhibitory polypeptide (GIP); (**c**) monocyte chemotactic protein-1 (MCP-1). Data are shown as the mean ± SD (*n* = 10). Different superscripts (*a*, *b*) indicate significant differences (*p* < 0.05).

Plasma leptin levels were significantly higher in both the C-HF and Q-HF groups than in the C-C group (Figure 5a). The plasma GIP level was remarkably increased in the C-HF group compared with the C-C group, but in the Q-HF group remained at a level comparable to that of the C-C group (Figure 5b). There were no differences in plasma MCP-1 levels among the groups (Figure 5c).

#### 3.2.4. Effects on Hepatic Lipid Levels

**Figure 6:**
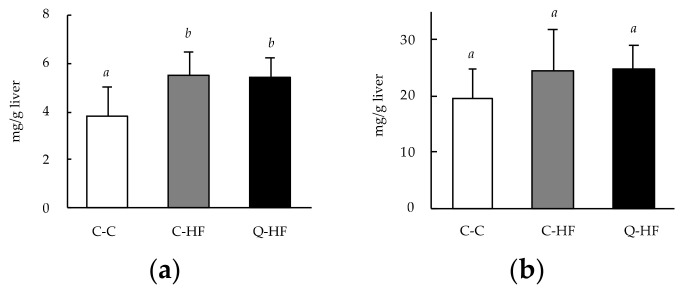
Effects of maternal quercetin consumption on hepatic total cholesterol and triglycerides. Male progeny of dams fed a control diet (C) or a diet containing 1.0% quercetin (Q) were divided into three groups: progeny of dams fed the C-diet that were also fed the C diet (C-C), progeny of dams fed the C-diet that were fed a 30% high-fat (HF) diet (C-HF), and progeny of dams fed Q-diet that were fed a HF diet (Q-HF). The animals were permitted free access to deionized water and the C-diet or HF-diet for 13 weeks, and then their hepatic levels of total cholesterol (**a**) and triglycerides (**b**). Data are shown as the mean ± SD (*n* = 10). Different superscripts indicate significant differences (*p* < 0.05). Different superscripts (*a*, *b*) indicate significant differences (*p* < 0.05).

Among the major hepatic lipids, total cholesterol levels were significantly higher in both the C-HF and the Q-HF group than in the C-C group (Figure 6a). However, there were no differences in hepatic triglycerides levels among the groups (Figure 6b).

## 4. Discussion

Obesity has become an important worldwide concern, especially in developed countries. In this study, we evaluated the effects of maternal quercetin consumption during pregnancy on obesity-related parameters in male progeny fed a HF diet. Maternal quercetin (1.0%) consumption during pregnancy did not affect the dams’ body weights (Table 2) or blood parameters, including total cholesterol, triglycerides, total protein, and glucose (Figure 2), compared with the control group at postnatal day 3. These results agree with our previous study, in which male mice were fed a diet containing 0.5% quercetin glucoside [12]. Maternal quercetin consumption also did not change the body weight of the neonates. Therefore, maternal consumption of quercetin in the context of the diet used in this study may not affect the body weight of dams or neonates.

Maternal quercetin consumption also did not affect the blood parameters in neonates. Although this is not one of the main findings from the study, blood triglyceride levels were significantly higher in neonates than in their dams (Figure 2b). McMullin et al. reported that triglyceride levels were 100-fold higher in breast milk than in maternal serum, favoring the accumulation of lipophilic chemicals in milk [26]. In this study, blood samples were collected from dams and neonates after 6 h of fasting. We did not isolate the neonates from the dams, in order to avoid inducing maternal separation stress [27], so the neonates could have obtained nutrients from breast milk even during the fasting period. Therefore, it is difficult to compare the data between the dams and neonates; however, our data indicate that neonates are exposed to high levels of triglycerides during critical stages of development via dams’ breast milk. Blood protein levels were significantly lower in neonates than in dams (Figure 2c). These results are similar to those reported by McMullin et al. [26], and suggest that blood protein levels in neonates may increase progressively to adult levels by postnatal day 20. Total cholesterol levels also lowered significantly in the neonates. A positive correlation between blood cholesterol and blood protein levels has been reported [28]; hence, the lower total cholesterol levels observed in the neonates could have been correlated with overall lower in blood protein levels.

Male mice are recognized to be more likely to become obese than female mice, and that the protection against obesity in female mice is eliminated by ovariectomy, indicating that there are metabolic differences between males and females [29]. However, many research groups, including us, have employed male mice, and evaluate the functional aspects of quercetin [9,11,12]. Therefore, we firstly focused on the male progeny, and evaluated the effects of maternal quercetin consumption during pregnancy on high-fat diet–induced obesity. Daily consumption of the HF-diet resulted in increased weight gain in the C-HF group compared with the C-C group (Figure 3). The C-HF group consumed less food on a gram-per-gram basis but a similar amount of overall energy compared with the C-C group (Table 3). Similar HF diet–induced obesity has been reported in mice models previously [9,30]. Maternal quercetin consumption (Q-HF) accelerated the HF-diet–induced weight gain compared with mice in the C-HF group, which had a similar food intake. The visceral fat weight in both groups fed the HF diet was higher than that of the C-C group, suggesting HF diet–induced obesity. Interestingly, liver weight was significantly increased in the Q-HF group compared with the C-C and C-HF groups (Table 3). Yamaki and Takahashi fed male C57BL/6 mice a diet containing 0.01–1.0% quercetin for 4 weeks and found that the liver weight was significantly increased only in the mice that received the 1.0% quercetin diet, even though their body weight did not change significantly compared with the control groups and the other quercetin groups [25]. We found that liver weight increased in the Q-HF group, although the progeny did not consume any quercetin during the experimental period. This result suggests that maternal consumption of a high amount of quercetin, such as 1.0% of the diet, could have hepatic effects in the next generation. Liver weight gain induced by consumption of a HF diet typically occurs due to hepatic lipid deposition [31]. However, we did not observe any differences in hepatic lipid levels, total cholesterol, or triglycerides between the C-HF and Q-HF groups (Figure 6). Therefore, it is unclear whether maternal quercetin consumption promotes toxic liver weight gain in progeny that consume a HF diet. Our data imply another hypothesis; whether such pro-obesogenic effects observed at Q-HF group (first generation) are reinforced on the next progeny from Q-HF group (second generation). This is our future topic.

Blood levels of total cholesterol and non–HDL-cholesterol were significantly increased in the C-HF group compared with the C-C group (Figure 4a,b). The Q-HF group exhibited a similar pattern, with total cholesterol levels even more significantly elevated compared with the C-C group than those seen in the C-HF group. These changes did not result from malnutrition, as there were no differences in food consumption (Table 3) or in the serum total protein level (Figure 4f), which is traditionally used as a marker for nutritional status [32]. An increase in blood LDL-cholesterol levels is a recognized indicator of metabolic disease, including obesity [33]. Therefore, the higher levels of total cholesterol and non-HDL cholesterol observed in the Q-HF group were recognized as abnormal. However, the level of HDL-cholesterol, which is considered to be beneficial, in the Q-HF group was significantly increased compared with the C-C and C-HF groups, which exhibited similar levels (Figure 4c). Recently, the total cholesterol/HDL-cholesterol ratio in the blood has been identified as a useful index for obesity [34]. Indeed, this ratio has been reported to be significantly higher in ICR mice with HF diet–induced obesity than in control mice [35]. This is in accordance with our results, which showed that the total cholesterol/HDL-cholesterol ratio in the C-HF group was significantly higher than in the C-C group, while the ratios were similar in the Q-HF and C-C groups (Figure 4d), in which levels agree with the published values [36,37]. These results clearly indicate the possibility that maternal quercetin consumption may protect the next generation from disruption of cholesterol metabolism by consumption of a HF-diet, even though total cholesterol itself will increase. On the other hand, some research groups have reported lower total cholesterol/HDL-cholesterol ratios compared with our result observed in the C-C group (around 10.3) in mice consuming control fat diet, for example, about 1.8 by Yu et al. [38]. Their results indicate similar total cholesterol amounts in blood (118 mg/dL) with our result (103 mg/dL), indicating that HDL-cholesterol values were remarkably different between their data (66.3 mg/dL) and our data (10.2 mg/dL). The reasons for such conflicting plasma HDL-cholesterol levels are unclear. However, some possibilities are indicated. Miller et al. reported that HDL-cholesterol levels resulted in more than 15% differences, when analyzing with eight kinds of commercial kits [39]. Additionally, handling methods such as fasting period, collection timing, and anesthesia might affect the blood biochemical parameters including total cholesterol and HDL-cholesterol levels [40]. Therefore, future studies will be required to check these points observed in the blood HDL-cholesterol levels on mice.

Consumption of a HF diet is known to increase blood leptin levels, which stimulate the hypothalamic centers that control food intake and energy expenditure to increase body fat stores [41]. In our study, blood leptin levels were significantly increased in the C-HF group compared with the C-C group, and maternal quercetin consumption did not alleviate this effect (Figure 5a). Consumption of a HF diet has also been reported to increase blood levels of GIP [42]. We observed the same effect in our study, and found that maternal quercetin consumption protected progeny from this HF-diet–induced elevation in blood GIP level (Figure 5b). GIP is secreted from duodenal endocrine K cells during stimulation by nutrients such as fats and glucose [43], and recognized to exert direct physiological effects on lipid metabolism [44]. We recently reported that daily consumption of quercetin glucoside reduces the blood levels of GIP [12]. Therefore, one of the putative mechanisms, that maternal quercetin consumption regulates cholesterol metabolism on their progeny, may be pass on regulation of GIP secretion from mothers to the next generation.

The advantage of using animal (mice) models to study metabolic syndrome, including obesity, is the ability to monitor functional and biochemical changes of metabolic syndrome, which is difficult to conduct in humans [45]. However, the animal dose should not be extrapolated to a human equivalent dose by a simple conversion based on body weight. Instead, a body surface area normalization method has been proposed to convert an animal dose to the equivalent human dose, which is often represented in mg/m^2^; the dose should be multiplied by the *K_m_* factor of 37 for adult humans and 3 for mouse [46]. For example, to convert the dose used in a mouse to dose for a human based on surface area, the daily 77 mg quercetin per 41.4 g body wight (Table 2) (about 1.8 g/kg) is multiplied by the *K_m_* factor of 3 for mouse and then divided by the *K_m_* factor of 37 for human adult. This calculation results in an adult human equivalent dose for quercetin of 0.15 g/kg, which equates to about 9 g quercetin for 60 kg person per day. This amount might be difficult for humans to daily consume from foods, because 9 g quercetin is calculated to be in about 15 kg quercetin-rich vegetable such as onion [47]. We have to continue the further studies using lower amounts of quercetin for maternal consumption.

## 5. Conclusions

In this study, we investigated whether maternal quercetin consumption during pregnancy protects their progeny from HF diet–induced obesity. Our data demonstrate that maternal consumption of quercetin may not affect body weight and blood lipid parameters in either dams or neonates at postnatal day 3. The Q-HF group exhibited overweight and increased liver weight, as well as higher blood cholesterol levels, compared with the C-HF group. However, the total cholesterol/HDL-cholesterol ratio remained similar to that observed in the C-C group. In conclusion, maternal quercetin consumption does not appear to protect the next generation from HF-diet–induced hyper cholesterol level in the blood and liver, and consequently overweight, but may help regulate the total cholesterol/HDL-cholesterol ratio in progeny. This study has some limitations, because of the insufficiency of some groups, for example, male progeny of dams fed Q-diet that were also fed the Q-diet, and also every female progeny. Therefore, more detailed studies should be undertaken in the future.

## Figures and Tables

**Table 1:** Composition of the AIN-93G–based experimental diets.

	Normal-Fat Diet		High-Fat Diet (HF)
	– (C)	Quercetin (Q)	
	in 100 g diets		
β-Cornstarch (g)	39.75	38.75	16.75
α-Cornstarch (g)	13.2	13.2	13.2
Casein (g)	20.0	20.0	20.0
Soybean oil (g)	7.0	7.0	7.0
Lard (g)	–	–	23.0
Sucrose (g)	10.0	10.0	10.0
Cellulose (g)	5.0	5.0	5.0
Vitamin mixture (g)	1.0	1.0	1.0
Mineral mixture (g)	3.5	3.5	3.5
L-Cysteine (g)	0.3	0.3	0.3
Choline bitartrate (g)	0.25	0.25	0.25
t-Butylhydroquinone (mg)	1.4	1.4	1.4
Quercetin (g)	–	1.0	–
Energy (kcal/g)	3.948	3.908	5.098

**Table 2:** Effects of quercetin consumption on food intake and body weight (first sub-group).

	Dams		Neonates	
	C-Diet	Q-Diet	C-Diet	Q-Diet
Body weight (g)	42.1 ± 4.1	41.4 ± 4.1	3.1 ± 1.0	3.3 ± 0.3
Number of animals per dam	5	6	12 ± 3	10 ± 3
Food intake (g/mice/day)	7.2 ± 1.1	7.7 ± 2.4	–	–
Quercetin intake (mg/mice/day)	–	77 ± 24	–	–

After acclimatization on the control (C) diet, the quercetin groups were switched to a diet containing 1.0% quercetin (Q). Breeding was carried out after 3 days on the Q-diet. The C-diet dams and their neonates, as well as the Q-diet dams and their neonates, were sacrificed on postnatal day 3 (first sub-group). Data are shown as the mean ± SD. There were no significant differences between the C-diet and Q-diet groups.

**Table 3:** Absolute and relative organ weights.

	C–C	C–HF	Q–HF	ANOVA *p* Values	
				Maternal Diet	Progeny Diet
Body weight					
Initial (g)	37.8 ± 2.9 *^a^*	38.7 ± 2.1 *^a^*	37.7 ± 2.9 *^a^*	0.582	0.747
Final (g)	54.6 ± 6.2 *^a^*	63.4 ± 4.5 *^b^*	71.3 ± 12.2 *^c^*	0.001	<0.001
Food consumption					
(g/mouse/day)	5.1	4.0	4.1	–	–
(kcal/mouse/day)	20.1	20.4	20.9	–	–
Absolute organ weight (g)					
Liver	2.18 ± 0.29 *^a^*	2.45 ± 0.36 *^a^*	3.56 ± 1.53 *^b^*	0.003	0.041
Kidney	0.85 ± 0.06 *^a^*	0.79 ± 0.08 *^a^*	0.77 ± 0.15 *^a^*	0.186	0.111
Spleen	0.21 ± 0.05 *^a^*	0.22 ± 0.07 *^a^*	0.20 ± 0.05 *^a^*	0.514	0.827
Heart	0.27 ± 0.03 *^a^*	0.27 ± 0.02 *^a^*	0.26 ± 0.03 *^a^*	0.221	0.411
Lung	0.25 ± 0.03 *^a^*	0.25 ± 0.02 *^a^*	0.25 ± 0.04 *^a^*	0.588	1.000
Visceral fat *	2.70 ± 1.21 *^a^*	4.23 ± 0.79 *^b^*	4.74 ± 1.37 *^b^*	0.012	<0.001

Data are shown as the mean ± SD (*n* = 10). * Epididymal fat + perirenal fat weights. Different superscripts (*a*, *b*, *c*) indicate significant differences (*p* < 0.05).

## Data Availability

No new data were created or analyzed in this study. Data sharing is not applicable to this article.
